# Generalized Dystonia Treated With Deep Brain Stimulator: An Institutional Single Surgeon Experience

**DOI:** 10.7759/cureus.10992

**Published:** 2020-10-16

**Authors:** Hammad Ghanchi, Jacob E Bernstein, Taha M Taka, Tye Patchana, Samir Kashyap, Omid R Hariri, Ali O Jamshidi, Ajay K Ananda

**Affiliations:** 1 Neurosurgery, Riverside University Health System Medical Center, Moreno Valley, USA; 2 Neurosurgery, University of California Riverside, Riverside, USA; 3 Neurosurgery, Kaiser Permanente-Orange County, Anaheim, USA; 4 Neurosurgery, Kaiser Permanente, Los Angeles, USA

**Keywords:** dystonia, deep brain stimulation

## Abstract

Introduction

Dystonia can cause severe disability when left untreated. Once a patient has exhausted medical management, surgical intervention may be the only treatment option. Although not curative, deep brain stimulation has been shown to be beneficial for patients affected by this condition. Our study sought to review patients undergoing deep brain stimulation for medically refractory dystonia to assess outcomes.

Methods

Our institution's operative database was reviewed retrospectively for all patients undergoing deep brain stimulator placement over the last six years. These medical records were reviewed for the severity of dystonia preoperatively and followed postoperatively for 24 months, focusing on the Burke-Fahn-Marsden Dystonia Rating Scale (BFMDRS). Patients with less than two-year postoperative follow-up were excluded from the study. The patients were further stratified by age into Group A, consisting of patients less than 40 years old, and Group B, patients greater than or equal to 40 years old. Other attributes such as age, sex, age of disease onset, disease duration at the time of surgery, genetic tests for dystonia-related genes, and any complication associated with surgery were also reviewed.

Results

Four hundred fifty-five operative cases for deep brain stimulator placement were reviewed, and 16 patients met inclusion criteria for the study. The mean age for our patient cohort was 43.75 years, with four males and 12 females. The average time from the age of disease onset to time of surgery was 9.7 years for Group A and 10.8 years for Group B; the overall average was 10.3 years. All patients had globus pallidus interna (GPi) as their surgical target. The first incidence of a statistically significant decrease in BFMDRS score was noted at three months postoperatively (p<0.001) when compared to preoperative values. Fourteen patients in our cohort underwent preoperative genetic testing for DYT gene mutations, out of which four were found to have a mutation.

Conclusion

Our review of outcomes for primary generalized dystonia at our institution found that deep brain stimulator targeting the GPi is safe and effective. We found an overall 88% response rate with younger patients (< 40-year-old) showing a better response at two years than older patients.

## Introduction

Dystonia is a neurological condition characterized by uncontrolled and sustained contractions of striated muscles leading to repetitive, involuntary muscle spasms with abnormal postures and movements without cognitive involvement [1]. Studies have reported the prevalence of dystonia to be 15-30 cases per 100,000; however, these studies acknowledge that this is likely an underestimate of the true prevalence of the disorder secondary to a wide array of presentations [1,2]. One study reports a prevalence of 732 per 100,000 in the general population over the age of 50, suggesting an increased prevalence in the elderly population [3]. Dystonia can be designated as primary versus secondary, early-onset versus late-onset, and focal versus segmental [4,5]. Early-onset dystonia begins in childhood and is often segmental or generalized, while late-onset dystonia occurs after age 25 and is typically focal involving the craniocervical muscles [4,5]. Secondary dystonia is a result of known pathological conditions, such as metabolic disorders, anoxia, trauma, drugs, or stroke. However, most cases of dystonia are classified as primary dystonia, either idiopathic or related to a known inheritance pattern.

Current treatments of dystonia can be divided into three main categories: (a) oral drug treatments, (b) localized botulinum toxin injections, and (c) deep brain stimulation (DBS). Oral drug treatments consist of levodopa to treat dopa-responsive dystonia [6]. Anticholinergic medications such as trihexyphenidyl are beneficial in pediatric-onset generalized dystonia, less effective in adult-onset focal dystonia, and is better tolerated in children than adults [7]. Botulinum toxin injections are an effective treatment of choice for patients with focal and segmental dystonia [8]. In cases where patients are unresponsive to oral treatments or their condition is complicated by generalized or complex segmental dystonia, DBS is recommended as a long-term treatment option; however, it is found to be the most effective in primary dystonia [9,10].

Bilateral DBS of the globus pallidus interna (GPi) has proven to be an effective treatment of primary segmental or generalized dystonia though less effective for secondary dystonia [9,11]. Despite the presence of other potential targets for DBS, such as the subthalamic nucleus (STN), DBS of GPi has been extensively studied as an effective therapeutic target in this condition. The study aimed to review our institution’s outcomes for patients with generalized dystonia undergoing deep brain stimulator placement.

## Materials and methods

Our institution's operative database was reviewed retrospectively to find patients with any diagnosis of dystonia refractory to medical therapy who underwent any deep brain stimulator placement. Operative logs were reviewed for all patients who underwent deep brain stimulator placement between 2014 and 2018 by the senior author surgeon (AA). These charts were then sorted by medical diagnosis code to identify patients with "dystonia" in their medical history. A further chart review was performed to find patients with primary generalized dystonia. Patients with less than a two-year follow-up were excluded from the study. A total of 16 patients were identified who met the inclusion criteria for our study. Patient attributes were collected with a focus on the Burke-Fahn-Marsden Dystonia Rating Scale (BFMDRS) at the preoperative visit, postoperative one-month, three-month, 12-month, and 24-month visits [12]. Other attributes, such as age, sex, age of disease onset, disease duration at the time of surgery, genetic tests for dystonia-related genes, and any complication associated with surgery, were also reviewed. The difference in the BFMDRS was calculated, comparing preoperative values to the consecutive postoperative values. The patients were further stratified by age into Group A, consisting of patients less than 40 years old, and Group B, patients greater than or equal to 40 years old. This age was chosen considering the modal distribution of dystonia. Statistical analysis was performed using Student's t-test to compare differences in BFMDRS values.

## Results

Among the 455 cases reviewed at our institution who underwent deep brain stimulator placement, a total of 16 patients met the inclusion criteria for our study. All patients were treated with bilateral GPi DBS electrodes for generalized dystonia. One patient required revision surgery secondary to wound infection at the implanted impulse generator site; this was treated without removing the deep brain stimulator, and the patient was included in the cohort. The same patient also started to have worsening of dystonia at the 24-month follow-up visit and was later treated with STN-targeted DBS outside the current study window. One patient had no significant improvement in dystonia and was later placed on comfort care measures following complications with his gastrostomy tube at 30 months postoperative from initial deep brain stimulator placement; this was outside the current study follow-up window, so the patient was included in our cohort. All cases were performed by the senior author (AA) with the assistance of a resident or midlevel provider. The mean age for our patient cohort was 43.75 years, with four males and 12 females. One patient was pediatric (age 14), with the remainder age 18 or greater at the time of surgery.

The average time from the age of symptom onset to the time of surgery was 10.3 years. The first incidence of a statistically significant decrease in BFMDRS score was noted at three months postoperatively (p<0.001) when compared with preoperative values. There was a statistically significant decrease in the disability subset of BFMDRS (BFMDRS-D) noted at the one-month visit (p=0.03) with the motor subset (BFMDRS-M) and total BFMDRS scores trending down without reaching statistical significance at this time. The change in BFMDRS values after the three-month visit continued to trend downwards but did not have a statistically significant decrease when compared with prior visit values (i.e., three-month to 12-month, 12-month to 24-month).

There were more females (n = 12) than males (n = 4) in our cohort, but there was no significant difference noted in BFMDRS values, or the subset BFMDRS-M and BFMDRS-D, between these two groups in the study window. Comparison of preoperative values to 24-month postoperative values yielded no significant difference as well between the sexes.

**Figure 1:**
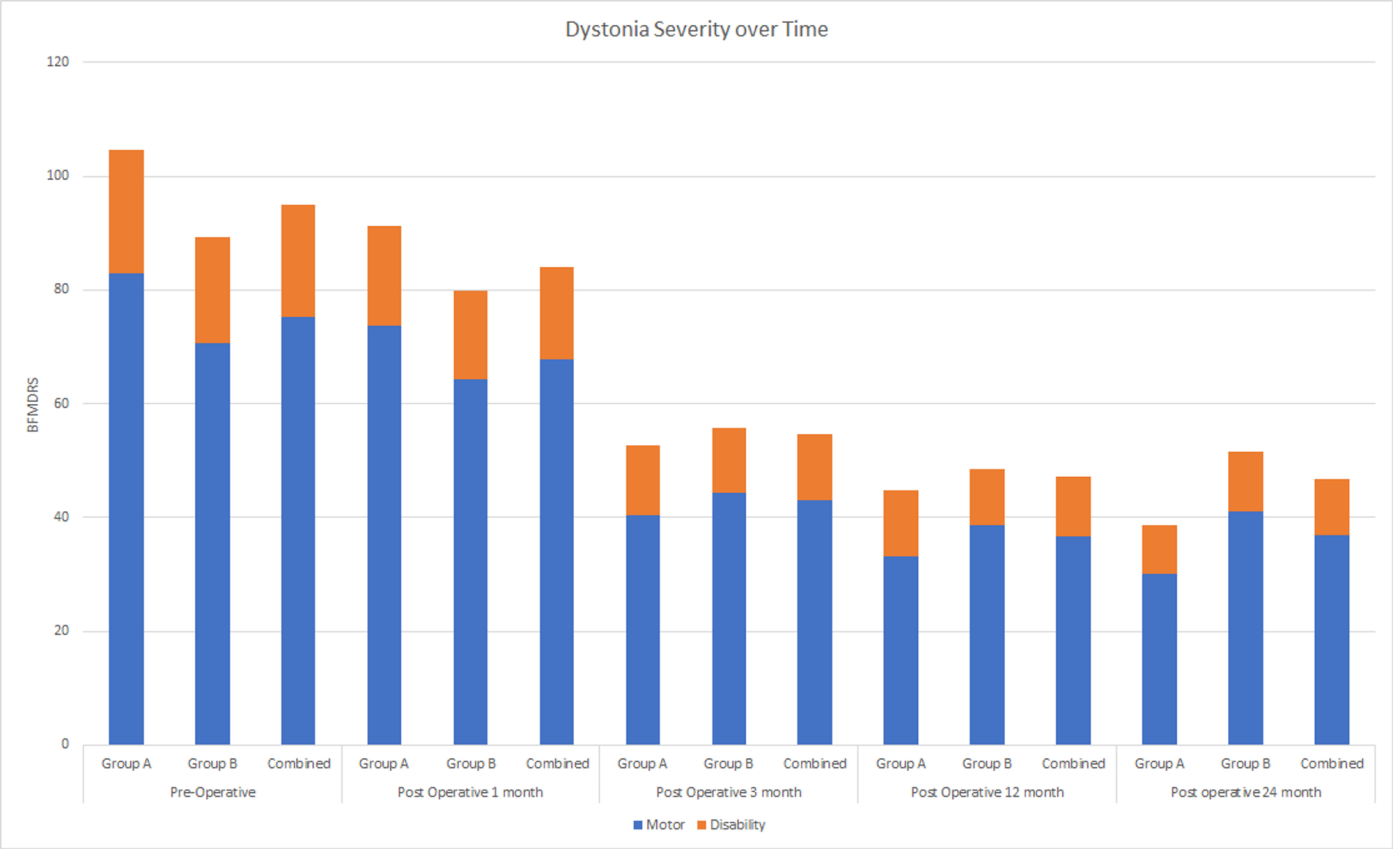
Dystonia Severity Over Time BFMDRS: Burke-Fahn-Marsden Dystonia Rating Scale

Patient data were further stratified by age given the typical bimodal distribution of dystonia in the population; Group A was less than 40 years old and Group B comprised patients who were 40 years or older. The average time from the age of disease onset to time of surgery was 9.7 years for Group A and 10.8 years for Group B; the overall average was 10.3 years for the cohort. There were no statistically significant differences noted between Group A and Group B when comparing BFMDRS-M, BFMDRS-D, and BFMDRS at each follow-up visit interval. Downward trend in scores continued throughout the study period, with the exception of Group B showing plateau results sooner (Figure 1). There was a significant difference noted when comparing the preoperative values with the 24-month postoperative values between Group A and Group B; Group A benefited more in terms of their BFMDRS (p<0.01) as well as subset BFMDRS-M (p<0.01) and BFMDRS-D (p<0.01) scores. Reduction in BFMDRS for each patient can be found in Table 1.

**Table 1:** Reduction in Dystonia Severity (Preop to 24 months Postop DBS) The table demonstrates the decrease in BFMDRS scores over the 24-month period for each patient. The breakdown of the BFMDRS motor and disability divisions is also demonstrated. Values were calculated by subtracting the postoperative score from respective preoperative scores. Preop: preoperative; Postop: postoperative; DBS: deep brain stimulation; BFMDRS: Burke-Fahn-Marsden Dystonia Rating Scale; Pat. No.: patient number; BFMDRS-M: Burke-Fahn-Marsden Dystonia Rating Scale motor division; BFMDRS-D: Burke-Fahn-Marsden Dystonia Rating Scale disability division; M: male; F: female

			Reduction in Dystonia Severity (Preop to 24 months Postop DBS)							Mean		
Pat. No.	Age	Sex	BFMDRS	BFMDRS-M	BFMDRS-D	BFMDRS	BFMDRS-M	BFMDRS-D		BFMDRS	BFMDRS-M	BFMDRS-D
1	14	M	70	56	14	58.33%	59.57%	53.85%	Group A	66 (63.38%)	53 (63.92%)	13 (61.49%)
2	18	F	69	54	15	65.09%	65.85%	62.50%				
3	20	F	92	76	16	64.79%	67.86%	53.33%				
4	20	F	54	42	12	67.50%	65.63%	75.00%				
5	22	F	47	38	9	65.28%	65.52%	64.29%				
6	33	F	64	52	12	59.26%	59.09%	60.00%				
7	43	M	45	34	11	50.00%	47.22%	61.11%	Group B	37.6 (43.09%)	29.6 (42.56%)	8 (45.19%)
8	44	F	37	32	5	51.39%	57.14%	31.25%				
9	44	M	10	8	2	9.80%	10.26%	8.33%				
10	55	F	31	24	7	48.44%	46.15%	58.33%				
11	56	F	53	42	11	55.21%	53.85%	61.11%				
12	59	F	36	28	8	43.90%	41.18%	57.14%				
13	65	F	46	40	6	39.66%	43.48%	25.00%				
14	65	M	36	26	10	47.37%	44.83%	55.56%				
15	70	F	39	26	13	45.35%	40.63%	59.09%				
16	72	F	43	36	7	39.81%	40.91%	35.00%				

The preoperative average BFMDRS score of Group A (104.7) was higher than Group B (82.9), with the final 24-month follow up average lower in Group A (38.7) than Group B (51.6). Moreover, the 24-month average of Group B begins to trend higher when compared to the 12-month average, whereas the values for Group A continue to decline (Figure 1).

Chart review for the history of genetic testing as etiology for dystonia was also performed. DBS has been shown to be more effective for certain gene mutations, particularly variants DYT1 gene mutations [10,13,14]. Fourteen patients in our cohort underwent preoperative genetic testing for DYT gene mutations. Out of these 14, four patients were found to be positive for a DYT gene mutation. Three of these patients were in Group A; two were positive for DYT1, and one was positive for DYT16. One patient in Group B was positive for DYT3. Furthermore, one patient in Group B had a PANK2 mutation, which is known to cause Hallervorden-Spatz Syndrome, or pantothenate kinase-associated neurodegeneration (PKAN) [15].

## Discussion

We found a statistically significant decrease in dystonia symptoms at three months after GPi deep brain stimulator placement when compared with preoperative status (p<0.001), consistent with the current literature. Unlike essential tremor or Parkinson's disease, the most common indications for DBS, the effects occur in a more delayed fashion, and the reason for this is unknown [16,17]. A possible mechanism was proposed that DBS increases output from the stimulated nucleus, GPi in this case, resulting in a complex pattern of excitatory and inhibitory effects on the basal ganglia thalamocortical (BGTC) network; this regulation prevents transmission of pathological bursts and oscillatory activity in the network [18,19]. However, no definitive evidence for how and why the delayed effects are seen exists to date. Another possible explanation for the delayed improvement is the need for optimization of DBS programming. Many patients require several programming sessions before the stimulation parameters are in an optimal setting. Additionally, the GPi is a much larger target than the STN and requires much higher stimulation settings. The higher charge density required may also result in delayed improvement given its size [20].

In our cohort, there was a significant change noted in the BFMDRS-D score at the one-month follow-up with a downtrending BFMDRS-M score and total BFMDRS score. This improvement in disability prior to a statistically significant decrease in motor may be contradictory to the presumption that motor should improve first. There may be a placebo effect at play, which explains patients being more ambitious and functionally independent after undergoing a surgical procedure, which they believe will help their condition. This may warrant researching into the psychological factors causing disability with dystonia.

**Figure 2:**
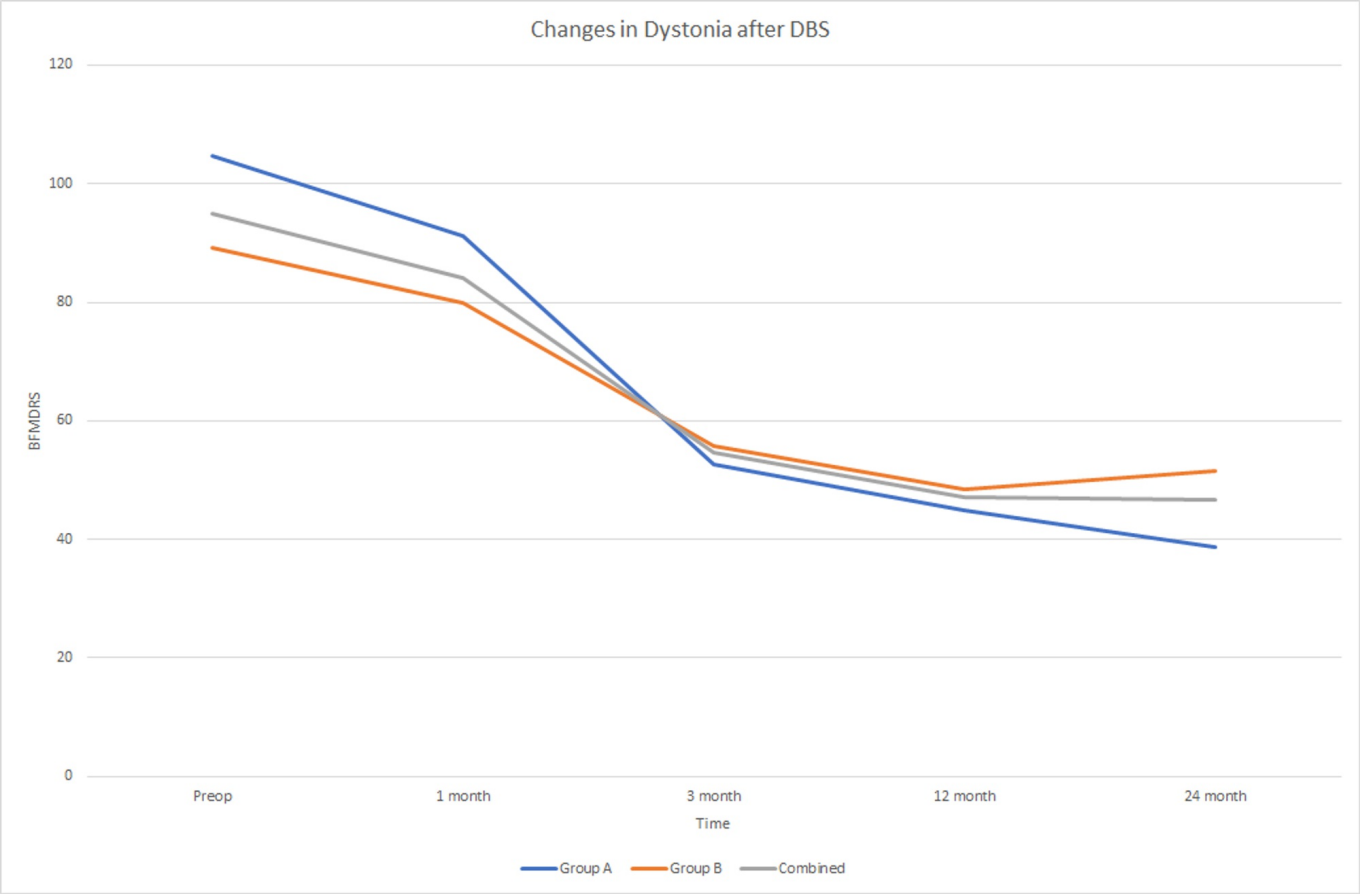
Changes in Dystonia After DBS DBS: deep brain stimulation; BFMDRS: Burke-Fahn-Marsden Dystonia Rating Scale

Group A had higher preoperative BFMDRS scores and lower 24-month postoperative scores than Group B (Figure 2). Although this difference was not statistically significant, age plays a factor in responsiveness to DBS for dystonia. Isaias et al. have demonstrated that younger patients with shorter disease duration have better outcomes after one year with deep brain stimulator placement [21]. Moreover, Group B had a slight increase in BRMDRS score at the 24-month follow-up compared with the 12-month follow-up. This increase was not statistically significant, and the exact reason for this remains unclear. The disease duration in our case was similar in both groups, 9.67 years and 10.8 years for A and B, respectively, so this is unlikely the cause. Moreover, given that Group A is still demonstrating a decrease in BFMDRS scores, a longer follow-up may be warranted for longitudinal analysis.

One patient had no significant response to DBS therapy (9.8% decrease in BFMDRS), and a second patient had a significant response (39.6% decrease in BFMDRS) but not to a level the patient would have liked and opted for a second surgery for STN-targeted DBS. If both patients are categorized as nonresponders in our cohort to GPi DBS, that will put our nonresponder rate at 12.5%. This is lower than the nonresponder rate in literature of around 25% [11,17,22]. Of note, both patients were in Group B with the ages of 44 and 65 at the time of surgery. Their preoperative BFMDRS scores were not the highest in our cohort. Moreover, Sun et al. have advocated for STN over GPi for generalized dystonia with improvement ranging from 76% to 100% in BFMRS scores in their series of 12 patients [23]. However, studies have reported similar results between the two nuclei with no significant difference [24] versus a slightly higher side effect profile for STN targeted patients [25].

Complication rate in our cohort was low, with no severe morbidity or mortality related to surgery. One patient (6%) developed a superficial wound infection, which was treated with local debridement without the need to explant the device. One patient (6%) was noted to have fevers postoperatively and was found to have a lower extremity thrombus (DVT) five days after surgery; this complication was likely related to their preoperative bedbound state due to dystonia rather than the surgery itself. There was no significant activity limitation after surgery, and the patient was more mobile in the postoperative period. One patient who did not respond significantly to deep brain stimulator placement later had a complication with his gastrostomy tube 30 months postoperatively; the family did not want further surgical treatment and opted for comfort-focused measures.

Mechanism of dystonia

Dystonia is thought to be caused by disruption of the normal function of the BGTC motor circuit, which is responsible for smooth and controlled motor movements. Under normal function, the BGTC motor circuit is composed of a hyperdirect (inhibitory) pathway, a direct (excitatory) pathway, and an indirect (inhibitory) pathway. The hyperdirect pathway will begin when the motor cortex excitatory signals activate the STN. This excitation will cause the STN to increase its excitatory signals to the GPi. The GPi normally functions to inhibit the downstream ventrolateral (VL) nucleus of the thalamus; therefore, its activation will cause increased inhibition of the VL nucleus of the thalamus. The direct pathway begins when the striatum, specifically the caudate and putamen nuclei, receive excitatory input from the motor cortex. When excited, these nuclei release gamma-aminobutyric acid (GABA) to inhibit the GPi, which in turn decreases GPi's inhibition of the thalamus. Ultimately, decrea­­sed inhibition of the thalamus causes overall excitation that is directed to the motor cortex resulting in increasing movement. Similarly, the indirect pathway also begins with the excitatory signals from the motor cortex to the striatal nuclei causing GABA release. However, GABA release from the striatum in the indirect pathway is targeted to the globus pallidus externus (GPe). GPe normally inhibits the STN; therefore, increased inhibition of GPe, in turn, causes excitation of the STN. Excitation of STN increases the stimulation of GPi, which, as discussed before, will increasingly inhibit the VL nucleus of the thalamus and decrease the excitement of the motor cortex [26]. In dystonia, there is increased inhibition of GPe, in the indirect pathway and GPi, in the direct pathway. Ultimately, this leads to decreased inhibition of the motor cortex movements and an increase in motor activity characterized by dystonia. 

The true mechanism of action of DBS on GPi remains unclear; however, its study has resulted in three different mechanistic hypotheses: the "inhibition hypothesis," the "excitation hypothesis," and "disruption hypothesis" [16]. As their names suggest, the inhibition hypothesis and excitation hypothesis are opposing theories that argue that DBS causes inhibition or excitation of local neuronal elements, respectively. However, both the inhibition and the excitation hypotheses fall short of a complete explanation of clinical findings. For example, many studies have shown t­he inability of the inhibition hypothesis to explain the clinical findings where DBS of GPi can treat dystonia, a condition caused by low-activity GPi [16]. Similarly, the opposing excitation hypothesis also faced difficulties since data has been presented that suggests inhibitions within stimulated sites. In a recent review, an alternative and well-supported "disruption hypothesis" was proposed [16]. In brief, the disruption hypothesis proposes that DBS causes disruption between the input and output of the targeted nucleus. This hypothesis works well to explain the effectiveness of DBS in dystonia, where abnormal information flow causes the manifestation of symptoms. ­­

Limitations

Given the retrospective nature of this study, it was difficult to standardize the evaluation of each patient to a single neurologist, i.e., the BFMDRS scores were not calculated by a single observer. The surgical technique was standardized, given a single attending neurosurgeon was performing all the procedures, and no significant modifications in technique were made over the timeframe of the study. Moreover, there was no control group who had a sham procedure performed, and this may be an interesting test for the future given the improvement in disability scores before motor scores in our cohort to see if the placebo effect was the cause for this improvement. However, given the effectiveness of DBS for dystonia, a sham procedure and placement of a nonfunctional lead and impulse generator may be unethical. An interesting idea for a prospective study would be to turn on the impulse generator in a delayed fashion.

## Conclusions

Indications for DBS are steadily growing; dystonia is the third most common indication for deep brain stimulator placement after essential tremor and Parkinson's disease. In our review of outcomes for primary generalized dystonia at our institution, we found that DBS of the GPi is safe and effective, which is in agreement with prior published studies. We achieved a high response rate, with younger patients showing a better response. Patients see a significant reduction in disability as soon as one month postoperatively and a more significant improvement in motor and disability scores at three months. Patients continue to improve steadily throughout the studied time period, with older patients plateauing after the first year, whereas younger patients continue to show improvement.
